# Trends and Determinants of Dementia-Related Mortality in Mexico, 2017–2023

**DOI:** 10.3390/epidemiologia7010014

**Published:** 2026-01-20

**Authors:** Dennis M. Lopez-Samayoa, Angel M. Campos-Sosa, Paola Asuncion Bojorquez-Chan, Sara E. Martinez-Medel, Jorge C. Guillermo-Herrera, Edgar Villarreal-Jimenez, Reinhard Janssen-Aguilar, Cristina Rodriguez Peres-Mitre, Nina Mendez-Dominguez

**Affiliations:** 1 School of Medicine, Universidad Autonoma de Durango, Durango 34200, Mexico; moshe56785678@gmail.com; 2 School of Medicine, Universidad Marista de Merida, Merida 97302, Mexico; angel.campos9117@gmail.com; 3 Centro Educativo Rodríguez Tamayo, Cd. Caucel, Merida 97314, Mexico; paola.bojorquez@certcaucel.edu.mx (P.A.B.-C.);; 4 Hospital Regional de Alta Especialidad de la Peninsula de Yucatan, Servicios de Salud del Instituto Mexicano del Seguro Social, IMSS-BIENESTAR, Merida 97130, Mexicocrodperezmitre@gmail.com (C.R.P.-M.); 5 Interventional Psychiatry Program, St. Michael’s Hospital, Toronto, ON M5B 1W8, Canada

**Keywords:** dementia, mortality, aging, Mexico, health disparities, ecological analysis

## Abstract

Background: Dementia is an increasing public health challenge in Mexico, yet recent national data on mortality patterns remain limited. This study examines temporal trends in dementia-related mortality and its sociodemographic and ecological characteristics among adults aged ≥65 years from 2017 to 2023. Methods: National mortality records from the General Directorate of Health Information were analyzed. Annual dementia-related mortality rates were calculated based on mid-year population estimates from CONAPO. Trends were assessed with regression analysis, including population offsets, and individual- and state-level characteristics were evaluated. Results: Between 2017 and 2023, dementia-related deaths increased from 761 to 1425, corresponding to an observed rise from 7.9 to 14.6 deaths per 100,000 inhabitants aged ≥65 years. Period trend indicated an average annual expected increase of 18.6% in dementia related mortality. A transient decline occurred in 2020–2021, coinciding with the COVID-19 pandemic. At the individual level, higher education was associated with greater odds of dementia certification, whereas Indigenous ethnicity appeared protective, which may reflect patterns consistent with diagnostic and reporting disparities. Higher state-level life expectancy correlated with higher dementia mortality, while greater population aging was inversely associated. Conclusions: Dementia-related mortality in Mexico shows a sustained upward trend with regional heterogeneity and apparent inequities in diagnosis and reporting. Strengthening mortality surveillance, improving certification quality, and integrating dementia indicators into national non-communicable disease registries are essential to guide equitable policy responses.

## 1. Introduction

Life expectancy is shaped by health care access, social determinants, and population health behaviors. While greater health care resources often extend longevity, system efficiency, education, and income inequality help explain why countries with similar expenditures may differ substantially in life expectancy [1,2]. Beyond spending, social protection, lifestyle factors, and satisfaction of health care needs are stronger determinants of survival [3].

However, increases in life expectancy do not always translate into a healthier life. A widening gap between life expectancy and health-adjusted life expectancy (HALE) has been documented, with many individuals spending additional years in poor health or disability [3,4,5,6]. This divergence underscores that prolonging life does not necessarily ensure quality of life. International studies, including those from Japan and China, confirm persistent disparities in healthy life expectancy despite health care expansion, largely influenced by resource distribution, socioeconomic status, and socioeconomic contextual factors [5,6,7,8].

In Mexico, life expectancy has risen in recent decades, yet autonomy and quality of life among older adults have not improved proportionally. Between 26.9% and 30.9% of older adults report some degree of dependency, and cognitive impairment is now a critical public health concern, affecting an estimated 1.3 million people with projections of 3.5 million by 2050 [9,10]. Dependency in later life results from multiple factors, such as chronic non-communicable diseases, such as diabetes and hypertension, sensory deficits, dementia, falls, and psychosocial factors, such as depression. Functional dependency is strongly linked to cognitive decline, particularly among women, individuals aged ≥75 years, and those with low education or limited resources [11].

Social determinants exacerbate vulnerability. Limited health care access, low socioeconomic status, and weak social networks are associated with functional dependency, poor self-rated health, and depression [12]. Many older Mexicans continue working beyond retirement age due to insufficient pensions, while gaps in universal coverage and persistent poverty deepen inequities [13,14], while indigenous identity intersects with health disadvantage, reflecting systemic barriers to care [14].

Recent political changes have shifted Mexico’s health system from a well-known yet imperfect insurance model to a potentially more inclusive system that remains under implementation. The transition from the known to the unknown is the moment we are living in today, and while some economists estimate that 80% of the population lacks health coverage, others anticipate a preventable crisis in highly specialized medical care. As a collateral effect of these changes, the elderly become more vulnerable and in need of specialized care for dementia [15,16,17], particularly because the public health system in Mexico is fragmented into subsystem institutions with profound differences in per-person health care budget, and deepening health inequities. Social determinants of health may affect an individual’s lifelong susceptibility to access health care and to cause-specific mortality; therefore, it is important to analyze individual characteristics related to dementia-related mortality and to identify national and state-clustered health indicators to help shape comprehensive strategies to mitigate dementia-related epidemiologic trends.

Cognitive impairment includes progressive neurodegenerative disorders such as Alzheimer’s disease, vascular dementia, and Parkinson’s disease dementia. Although aging is a major risk factor, dementia is not a normal consequence of aging. According to the National Population Council (CONAPO), adults aged ≥65 years represent about 9% of Mexico’s population in 2025, projected to reach 25% by 2050 [18]. Prior analyses identified higher dementia-related mortality among women, the oldest-old, urban residents, those with higher education, and individuals without a partner [19]. Dementia-related mortality in Mexico has not been sufficiently explained, given the context of population aging and changing health insurance coverage. Ecological analysis has proved useful for dementia-related mortality trend description [20].

### Study Aim and Hypotheses

This study aims to evaluate annual trends in dementia-related mortality in Mexico from 2017 to 2023, as well as the association of these trends with population aging, life expectancy, and health service affiliation at the state level. We hypothesized that (1) individual analysis may bring light to determinants for dementia-related mortality, (2) dementia-related mortality increased annually, with a possible dip during the COVID-19 pandemic years, (3) states with a greater aging population would also show higher dementia-related mortality, and (4) states may exhibit variability in health insurance coverage, which is relevant for dementia-related care.

## 2. Materials and Methods

### 2.1. Study Design

We conducted an ecological study integrating both individual and state-cluster analyses to examine dementia-related mortality in Mexico.

At the individual level, we analyzed national death certificate data to identify sociodemographic factors associated with dementia-related mortality. To identify the mortality cases in Mexico, records were obtained from the National Institute of Statistics and Geography (INEGI 2017–2023). We included all registries of deaths of individuals aged ≥65 with basic diagnosis coded as F00–F03X in ICD-10. Records of the foreign residence of the deceased were excluded.

Individual-level variables were age (as both, continuous and categorized by group: 65–79, 80–99, ≥100), gender (male, female), marital status (if living with a sentimental partner or not), basic education level (≥high school or <high school) economic activity (active vs. inactive), medical insurance (yes/no), area of residence (urban vs. rural), town size (<500,000 vs. ≥500,000 inhabitants), and Indigenous ethnicity (yes/no).

For state-clusters, we used state indicators as variables; we included indicators of aging, defined as the percentage of the population aged ≥65 years old, life expectancy at birth (in years), and percentage of population aged >65 with health insurance coverage, using data from INEGI and CONAPO open access databases. At the population level, each state of residence constituted a cluster, yielding 32 clusters.

### 2.2. Statistical Analysis

Descriptive statistics summarize individual characteristics; numerical variables are presented as means and standard deviations (SD), and for nominal variables, frequencies and percentages. For state-clusters, numerical variables are presented in medians and interquartile ranges (IQR; 25–75).

Standardized mortality rates per 100,000 inhabitants aged ≥65 years were estimated using the annual mid-year population projections for individuals ≥65 years, derived from the CONAPO. Trends were estimated using projected and observed mortality during pandemic years (2020–2021) using log-linear Poisson estimation (E[Deathst] = Pt × exp (β0 + β1 × Yeart).

For the individual analysis, a binary logistic regression was performed to identify the association between sociodemographic characteristics and the population size of the deceased’s place of residence (<500,000 vs. ≥500,000 inhabitants), expressed as Odds Ratios (ORs), 95% Confidence Intervals (CIs), and significance levels.

For the state-cluster analysis, given the nature of the data, accounting for a count, discontinuous numeric variable, non-parametric regression was developed to estimate associations.

All analyses were conducted using Stata^®^ 14.0, with statistical significance set at *p* < 0.05; graphics were performed with Excel using add-ins.

### 2.3. Ethical Considerations

This study used publicly available, de-identified secondary data. Institutional approval was obtained from the Research Board of the Hospital Regional de Alta Especialidad de la Peninsula de Yucatan, with approval number 2024-033.

## 3. Results

1. Annual Trends in Dementia-Related Mortality (2017–2023)

**Figure 1:**
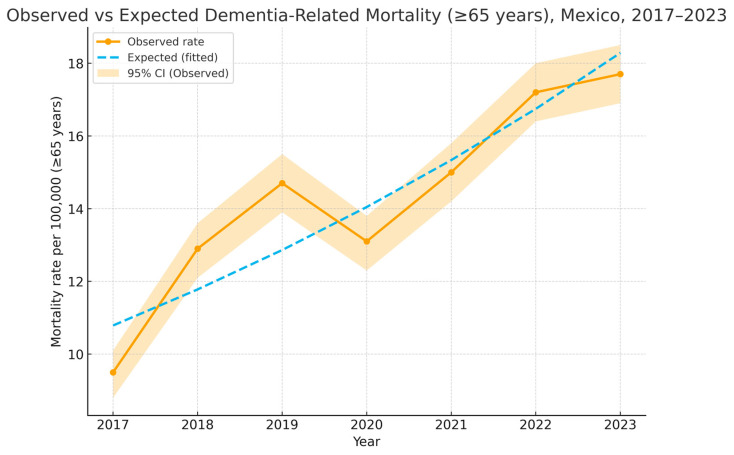
Observed rates were calculated using annual death counts attributed to dementia and mid-year population estimates for adults aged ≥65 years obtained from CONAPO. Expected rates were derived from a log-linear Poisson regression model fitted to 2017 to 2019 data, assuming exponential growth (annual expected increase = 18.6%). A transient decline was noted in 2020 to 2022, coinciding with the COVID-19 pandemic, followed by partial recovery in 2023.

A total of 8077 dementia-related deaths were registered among individuals aged ≥65 years between 2017 and 2023; annual deaths increased from 761 in 2017 to 1425 in 2023, with a transient decline in 2020 (1052 deaths), coinciding with the COVID-19 pandemic. Nationwide mortality rates per 100,000 inhabitants ≥65 showed a significant annual increase according to the expected (Poisson log-linear) trend by about 18–19% per year during 2017–2023. If the pre-pandemic pattern continued uninterrupted, by contrast, the observed year-over-year change averaged 11–12%, reflecting the transient slowdown around 2020–2022. Figure 1 shows observed and predicted mortality by year, illustrating an upward trend with a dip in 2020.

2. Individual-Level Sociodemographic Associations

At the individual level, we found that 60.6% of all dementia-related deaths were reported in women. The mean age at death was 85.4 (SD 7.7) years, which rose gradually from 84.4 to 85.2 years (*p* < 0.001), suggesting gains in longevity even among those dying with dementia; their demographic and social attributes can be found in Table 1.

Education patterns also changed modestly over time, as the proportion of individuals with at least elementary schooling increased across the studied period. In contrast, individuals cohabitating with a spouse declined slightly, while economic inactivity became more common. Medical insurance affiliation exhibited a decreasing trend, and proportionally more dementia-related deaths occurred among those without medical insurance. The proportion of individuals identified as indigenous remained relatively low across all years. The percentage of individuals living in a town with a population of less than 500 thousand inhabitants was 58.6%.

Binary logistic regression (Table 2) was performed to associate between individuals who resided in larger cities and socioeconomic determinants, showing that individuals from larger cities exhibited increased odds for elementary education or higher (OR 1.91, *p* < 0.001) and for medical insurance affiliation (2.22, *p* < 0.001) when compared to individuals residing in smaller cities or rural areas.

Conversely, belonging to an Indigenous group was less common among individuals residing in larger cities (OR 0.17, *p* < 0.001), and being economically active (OR 0.62, *p* < 0.001) was less likely among individuals residing in larger cities.

3. State-Clusters Analysis

**Figure 2:**
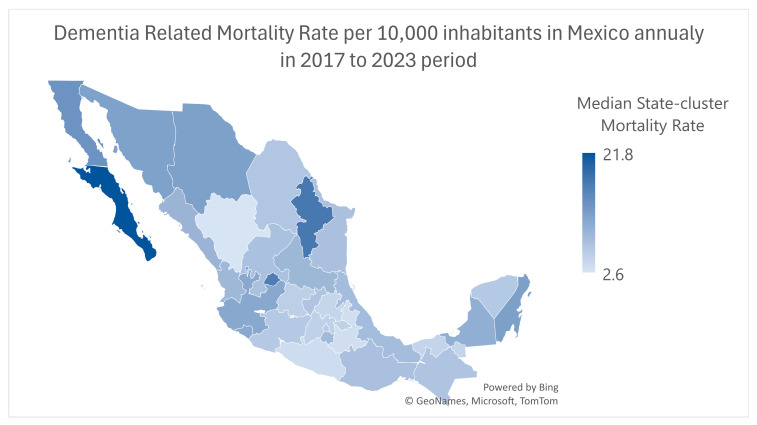
The state-by-state comparative chart shows dementia mortality rates in the 2017 to 2023 period for all 32 states.

Dementia-related mortality among adults aged 65 and over exhibited variability across the 32 state-clusters. Baja California and Nuevo Leon exhibited higher median rates in the studied period (Figure 1). Overall interstate variability can be found in Figure 2.

In Mexico, the median percentage of the population aging was 45.5 (IQR = 7.8); the median life expectancy was 75.2 (IQR = 2.1); and the median Medical Insurance coverage was 14.2 (IQR = 14.4). To determine state-cluster-level indicators pertaining to dementia-related mortality, we used a non-parametric regression with the percentage of the population aging, life expectancy, and the percentage of the population with health insurance as independent variables. In the multivariate regression model (Table 3), life expectancy emerged as a robust predictor, with each additional year associated with a 2.49-point increase in dementia mortality (*p* = 0.003). Population aging showed an inverse association (Coefficient = −0.21, *p* = 0.016), suggesting that states with proportionally larger older populations do not necessarily experience higher dementia mortality rates. This finding may reflect differences in diagnostic practices, reporting, or competing mortality risks.

A higher life expectancy was significantly associated with increased dementia-related mortality, whereas population aging showed a modest but significant negative association. Medical insurance coverage was not significantly associated with mortality variation across states.

## 4. Discussion

The present study analyzed national trends in dementia-related mortality in Mexico from 2017 to 2023, combining individual-level sociodemographic information with ecological indicators at the state level. The findings demonstrate a steady annual increase in dementia mortality, with a temporary decline in 2020 coinciding with the COVID-19 pandemic. Sociodemographic disparities were observed at both levels of analysis, including paradoxical associations that likely reflect differences in diagnosis, reporting, and survival patterns.

Our results from the present study showed an annual increase in dementia-related mortality among adults ≥ 65 years, even after adjusting for the pandemic years. This aligns with international reports of rising dementia burden, driven by population aging and increased recognition of cognitive impairment [1,21,22]. The observed decline in 2020 is consistent with pandemic-related disruptions: dementia may have been under-recorded on death certificates as COVID-19 dominated clinical care and vital statistics [23,24]. Similar patterns of underestimation during the pandemic have been reported in the United States, Spain, and Latin America [25,26].

At the individual level, dementia-related mortality occurred predominantly in women and in the oldest-old, consistent with global evidence of sex differences in dementia incidence and survival [27,28,29]. The gradual increase in age at death suggests improvements in overall survival, echoing trends documented in high-income countries [30].

Certain findings from the present study appear paradoxical. For example, a higher educational attainment was associated with greater odds for dementia-related mortality in larger cities, and despite education being a well-established protective factor against cognitive decline [31], it is also considered that more educated cohorts live longer, increasing their likelihood of dying later, when dementia mortality becomes more frequent. It may also indicate greater diagnostic accuracy in more educated individuals, as physicians may more readily evidence and record dementia on death certificates.

We found that economic activity and indigenous ethnicity were protective variables in larger cities when compared to smaller cities or rural areas, but this finding is unlikely to represent true protection; rather, it may suggest a lower proportional diagnosis in urban areas for indigenous population which may involve systemic underdiagnosis, cultural barriers, and underreporting in marginalized populations, consistent with prior work in Mexico and other Latin American countries [32,33]. Economically inactive individuals may have less access to medical insurance, which may explain the lower odds in larger cities compared to smaller ones. These disparities emphasize the need for culturally appropriate diagnostic approaches and improved access to specialized care in underserved regions.

At the state-cluster level, states with longer life expectancy reported higher dementia mortality, consistent with the demographic transition theory claiming that populations that survive longer inevitably face greater dementia burden [34,35]. However, states with a higher proportion of older adults did not necessarily show higher mortality. This counterintuitive result may reflect competing mortality risks in less developed regions, where older adults die earlier of other causes, reducing the probability of dementia being recorded as a basic cause of death [36].

Insurance coverage was not significantly associated with dementia mortality at the state level, despite being protective at the individual level. This discrepancy underscores the ecological fallacy, where population-level measures do not capture individual realities. It also suggests that affiliation alone may not guarantee timely diagnosis or adequate dementia care, as Mexico’s fragmented health system faces persistent inequities in service quality and access for the most vulnerable population [37,38,39].

The present study provides a comprehensive, annual national analysis of dementia-related mortality in Mexico, integrating both microdata and ecological indicators. It addresses prior gaps by disentangling individual and population-level associations and correcting for temporal trends. Our findings underscore the urgent need to strengthen dementia surveillance and care in Mexico. Improved diagnostic accuracy, particularly in underserved populations, is critical for reliable monitoring. Policies addressing the growing dementia burden must consider both demographic pressures (increasing life expectancy) and structural inequities (limited access in Indigenous and rural communities). Integrated approaches that combine public health, clinical care, and social support are necessary to ensure equitable management of dementia across Mexico.

The fact that health care affiliation does not correlate proportionally correlate to the population aging or the increase in life expectancy suggests, that if no systematic changes in health care coverage are implemented, with the continuous increase in life expectancy, population aging and dementia, Mexican adult population may face unmet needs for dementia related care, medical attention for the elderly in variable proportion, dependent on sociodemographic determinants at the individual and collective level. Policies for integrating health care resources and allocating them in equal manner to the aging population are needed, along with a collective sense of empathy and humanity to endorse and promote public policies for caring for the elderly.

### Limitation

Dementia seemingly remains underdiagnosed and underreported on death certificates, especially in rural populations and for underserved human groups. We based our study on ICD-10 codes and therefore could not distinguish between dementia subtypes, limiting etiologic insights. Even when the mortality rate provides an epidemiological measure of dementia, it is unclear whether the prevalence and real burden of the disease in Mexico; future studies may address this gap. Finally, state-cluster analyses may be interpreted with caution, to avoid precluding direct inference from state-level patterns to individual risk.

## 5. Conclusions

Dementia-related mortality in Mexico rose continuously between 2017 and 2023 nationally, with an expected annual increase of 18.6% and upward trends were observed in every state. Previous studies did not provide individual-level data alongside state-cluster correlates. This increase reflects both demographic changes associated with population aging and improved recognition of dementia in clinical and vital statistics. At the individual level, disparities were evident, including paradoxical associations such as higher risk among more educated groups and lower risk among Indigenous populations, likely reflecting differences in diagnostic practices, reporting, and survival. At the state-cluster level, longer life expectancy was a robust predictor of dementia mortality. In contrast, population aging and insurance coverage did not correlate, underscoring the complexity of structural influences and the need for a robust, inclusive health system to support the aging population equally.

These findings underscore the importance of developing integrated and equitable strategies for dementia surveillance and care. Strengthening dementia surveillance, improving diagnostic accuracy in underserved populations, and addressing inequities in access to health care and social support are critical to prepare Mexico for the growing dementia burden. National strategies must integrate demographic realities with targeted interventions to ensure equitable recognition, treatment, and care for older adults living with dementia.

## Figures and Tables

**Table 1:** Sociodemographic characteristics of dementia-related deaths in Mexico, 2017 to 2023, in the population aged ≥65 years.

Year Studied	2017	2018	2019	2020	2021	2022	2023
Frequency	761	1035	1184	1052	1211	1383	1425
Age (Mean, SD)	85.4 (7.8)	85.6 (7.7)	85.3 (7.6)	85.7 (7.5)	84.8 (7.5)	85.4 (7.8)	85.7 (7.6)
Male (%)	317 (41.8)	411 (39.9)	467 (39.8)	425 (40.4)	458 (37.7)	506 (36.9)	567 (40.0)
Indigenous ethnicity (%)	51 (6.7)	77 (7.3)	76 (6.4)	58 (5.5)	141 (13.3)	81 (5.8)	81 (5.7)
≥Elementary education (%)	100 (13.1)	149 (14.4)	171 (14.4)	176 (16.7)	192 (15.8)	240 (17.3)	279 (19.4)
Economically active (%)	192 (25.2)	349 (33.4)	264 (22.2)	237 (22.5)	270 (22.1)	284 (20.5)	307 (21.5)
Residence < 500 k people (%)	463 (60.7)	608(58.4)	687 (57.7)	651 (61.8)	714 (58.9)	814 (58.6)	797 (55.6)
Cohabiting with spouse (%)	222 (29)	309 (29.8)	351 (29.6)	300 (28.5)	343 (28.4)	364 (26.3)	387 (26.9)
Medical insurance (%)	646 (85.0)	883 (85.5)	992 (83.8)	798 (75.9)	847 (70.0)	933 (67.8)	977 (68.8)

Demographic and social attributes of decedents with dementia recorded nationwide from 2017 to 2023. Values represent counts with percentages in parentheses unless otherwise specified. Age is presented as mean ± standard deviation. Data indicate a gradual increase in total deaths, a predominance of female decedents, and a rise in educational attainment over time.

**Table 2:** Logistic regression of sociodemographic factors associated with town size of residence among dementia-related deaths in Mexico.

Sociodemographic Characteristic	Odds Ratio	z	*p*-Value	95% Confidence Interval	
				Lower	Upper
Age (numeric, per year)	1.00	0.08	0.934	0.99	1.00
65–79 years	0.89	−2.07	0.038	0.80	0.99
80–99 years	1.11	2.07	0.039	1.00	1.32
≥100 years	0.97	−0.18	0.859	0.71	1.32
Gender (Male)	0.78	−5.33	<0.001	0.71	0.85
Economically active	0.62	−8.41	<0.001	0.56	0.70
High school or higher	1.91	10.69	<0.001	1.70	2.16
Cohabiting with a spouse	0.99	-0.10	0.919	0.90	1.09
Medical insurance covers	2.22	14.13	0.001	1.98	2.48
Indigenous ethnicity	0.17	−13.29	<0.001	0.13	0.22

Logistic regression of sociodemographic factors associated with the place of death in smaller cities (<500,000 inhabitants) among dementia-related deaths in Mexico, 2017–2023 (population aged ≥65 years).

**Table 3:** Non-parametric Poisson regression reporting coefficients between state-cluster dementia related mortality rate and statewide indicators for the 2017–2023 period and population aged ≥65 years.

State/Cluster Characteristic	Coefficient	*p*-Value	95% Confidence Intervals	
			Lower	Upper
Life expectancy at birth	2.49	0.003	0.94	4.05
Population aging	−0.21	0.016	−0.38	−0.04
Medical Insurance Coverage	0.02	0.877	−0.29	0.34
Pseudo R2 = 0.42	Post hoc = 0.95			

Non-parametric Poisson regression coefficients for the association between state-level dementia-related mortality rates and statewide demographic and health indicators in Mexico, 2017–2023, for the population aged ≥65 years.

## Data Availability

The original data presented in the study are openly available at https://www.inegi.org.mx/programas/edr/#datos_abiertos (accessed on 12 July 2025).

## Abbreviations

The following abbreviations are used in this manuscript:

HALE	Health-adjusted life expectancy
YLD	Years lived with disability
DALY	Disability-adjusted life-years
CONAPO	National Population Council
INEGI	National Institute of Geography and Statistics
OR	Odds ratios
CI	Confidence intervals
